# A novel, integrated in vitro carcinogenicity test to identify genotoxic and non-genotoxic carcinogens using human lymphoblastoid cells

**DOI:** 10.1007/s00204-017-2102-y

**Published:** 2017-11-06

**Authors:** Eleanor C. Wilde, Katherine E. Chapman, Leanne M. Stannard, Anna L. Seager, Katja Brüsehafer, Ume-Kulsoom Shah, James A. Tonkin, M. Rowan Brown, Jatin R. Verma, Ann T. Doherty, George E. Johnson, Shareen H. Doak, Gareth J. S. Jenkins

**Affiliations:** 1 In Vitro Toxicology Group, Institute of Life Science 1, Singleton Campus, Swansea University Medical School, Swansea University, Swansea, SA2 8PP UK; 2 College of Engineering, Bay Campus, Swansea University, Swansea, SA1 8EN UK; 3 AstraZeneca, Discovery Safety, DSM, Darwin Building, Cambridge Science Park, Milton Road, Cambridge, CB4 0WG UK

**Keywords:** Carcinogenesis, In vitro, Genotoxicity, Multiple-endpoint, Carcinogenicity testing

## Abstract

Human exposure to carcinogens occurs via a plethora of environmental sources, with 70–90% of cancers caused by extrinsic factors. Aberrant phenotypes induced by such carcinogenic agents may provide universal biomarkers for cancer causation. Both current in vitro genotoxicity tests and the animal-testing paradigm in human cancer risk assessment fail to accurately represent and predict whether a chemical causes human carcinogenesis. The study aimed to establish whether the integrated analysis of multiple cellular endpoints related to the Hallmarks of Cancer could advance in vitro carcinogenicity assessment. Human lymphoblastoid cells (TK6, MCL-5) were treated for either 4 or 23 h with 8 known in vivo carcinogens, with doses up to 50% Relative Population Doubling (maximum 66.6 mM). The adverse effects of carcinogens on wide-ranging aspects of cellular health were quantified using several approaches; these included chromosome damage, cell signalling, cell morphology, cell-cycle dynamics and bioenergetic perturbations. Cell morphology and gene expression alterations proved particularly sensitive for environmental carcinogen identification. Composite scores for the carcinogens’ adverse effects revealed that this approach could identify both DNA-reactive and non-DNA reactive carcinogens in vitro. The richer datasets generated proved that the holistic evaluation of integrated phenotypic alterations is valuable for effective in vitro risk assessment, while also supporting animal test replacement. Crucially, the study offers valuable insights into the mechanisms of human carcinogenesis resulting from exposure to chemicals that humans are likely to encounter in their environment. Such an understanding of cancer induction via environmental agents is essential for cancer prevention.

**Electronic supplementary material:**

The online version of this article (10.1007/s00204-017-2102-y) contains supplementary material, which is available to authorized users.

## Introduction

Cancer is the second leading cause of mortality worldwide, with the number of new cases projected to rise by 70% over the next two decades (Stewart and Wild 2017). It has been demonstrated that 70–90% of human cancers are induced via exposure to environmental agents (Wu et al. 2016). Common routes of exposure to chemical carcinogens include the consumption of alcoholic beverages, tobacco smoking and occupational exposure.

Cancer may be initiated via both genotoxic and non-genotoxic mechanisms (Hanahan and Weinberg 2000, 2011). Most identified carcinogens fall within the initial group of genotoxic carcinogens (GCs), these triggering DNA mutation or chromosomal aberration (Hernandez et al. 2009). However, non-genotoxic carcinogens (NGCs), which constitute 10–20% of carcinogens (Bartsch and Malaveille 1989), demonstrate broader mechanistic variety, altering epigenetics, the endocrine system, apoptotic signalling, cell proliferation, and/or gap-junctional intercellular communication (Melnick et al. 1996; Uehara et al. 2008; Williams 2001). Furthermore, simultaneous alteration of multiple pathways is often required to prompt non-genotoxic oncogenesis (Guyton et al. 2009). Therefore, to understand an unknown carcinogenic mechanism, whether genotoxic or non-genotoxic, multiple-endpoint analysis is required. The eventual result, cancer development, combines uncontrolled cellular proliferation with genome instability, angiogenesis, and metastasis to distant tissues. Such characteristics have been defined as “Hallmarks of Cancer” (Hanahan and Weinberg 2000).

Carcinogenicity testing is a crucial aspect of compound development and safety assessment in pharmaceutical, food and agricultural industries. Such testing includes short-term in vitro assays, short-term in vivo assays, and the 2-year rodent bioassay (Kirkland et al. 2005). Banning of in vivo cosmetics testing in 2013 has increased dependence on in vitro tests, contributing to expense, time and ethical benefits. It is argued, particularly as part of Toxicity Testing in the 21st Century (Adeleye et al. 2015; Council 2007), that the in vitro shift may also improve human relevance: animal models often fail to represent human physiology, genetics and metabolism (Long 2007). Furthermore, recognition of the importance of the 3Rs (Reduction, Replacement and Refinement of animals in research) Principle is increasing. Development of more sophisticated in vitro assays is, therefore, key to future compound development.

Genotoxicity assays represent preliminary carcinogenicity testing, with the standard in vitro genotoxicity battery including the Ames test, micronucleus assay and the chromosomal aberration assay (Muller et al. 1999). Despite this battery achieving high sensitivity, factors such as variation between cell lines, time points, and incomplete compound metabolism reduce the specificity of results (Kirkland et al. 2005). An additional inadequacy of in vitro carcinogenicity assessment is the lack of approved tests for the identification of non-genotoxic carcinogens. For example, one currently available approach is the use of Cell Transformation Assays (CTAs), which utilises the phenotypic transformation of stem cells as a marker of carcinogenicity (Kerckaert et al. 1996). However, disadvantages include these assays’ subjectivity, qualitative results and lack of mechanistic insight. Cells used are often derived from rodent embryos (e.g., Syrian hamster embryo, mouse BALBc 3T3 and C3H/10T cells), and so it is unclear whether these tests can be considered to be true in vitro tests, resulting in 3Rs-related implications. Therefore, it is clear that more informative in vitro tests with greater specificity are urgently required.

The objective of this study was to improve the in vitro-based detection of carcinogenic mechanisms, including differentiation between GCs and NGCs by combining multiple cellular and molecular endpoints.

The test compounds were selected for their broad range of carcinogenic mechanisms (Table 1), allowing the applicability of the approach for identifying carcinogens to be assessed. The genotoxicity and mutagenicity of alkylating agents methyl methanesulfonate (MMS) and *N*-methyl-*N*-nitrosourea (MNU) is well established, with these producing differing profiles of methyl DNA adducts (Beranek 1990; Doak et al. 2007). Pro-oxidant hydrogen peroxide (H_2_O_2_) produces lesions such as 8-oxoguanine (Finnegan et al. 2010), whereas acetaldehyde induces lesions such as *N*2-ethyl-2′-deoxyguanosine (Brooks and Theruvathu 2005).

**Table 1:** A summary of the sources and mechanisms of the GCs and NGCs used in this study

Group	Compound	Sources of exposure/application	Primary mechanisms
GCs	Hydrogen Peroxide (H_2_O_2_)	Widely used as a disinfectant, antiseptic and oxidiser (Pinkernell et al. 1997). Hair dye ingredient (Kim et al. 2016)	A free hydroxyl radical which can react with DNA to produce lesions such as 8-oxoguanine (Finnegan et al. 2010)
	Acetaldehyde	Exposure through alcoholic beverages, cigarette smoke and used as an intermediate in chemical synthesis (Brooks and Theruvathu 2005)	DNA reactive; Sister chromatid exchange and chromosomal aberrations (Brooks and Theruvathu 2005)
	Methyl methanesulfonate (MMS)	Used in laboratory research as a solvent catalyst and potent model genotoxicant (HSDB 2000). Chemotherapeutic agent	DNA methylation primarily forming adducts such as 7-Methylguanine and 3-Methyladenine (Beranek 1990)
	*N*-methyl-*N*-nitrosourea (MNU)	Previously used as a precursor to diazomethane (Lijinsky 1992), now used in laboratory research	DNA methylation forming adducts 7-Methylguanine 3-Methyladenine, *O* ^6^-Methylguanine and an inducer of oxidative stress (Beranek 1990b)
NGCs	Bis-2-ethylhexyl phthalate (DEHP)	A key ingredient in the manufacture of poly-vinyl chloride medical plastics (Sampson and de Korte 2011)	Disruption to gap junctional intercellular communication (Melnick et al. 1996), oxidative stress (Rusyn et al. 2006) and activation of Aryl Hydrocarbon (Ah) receptor (Kruger et al. 2008)
	Methyl carbamate (MC)	An intermediate in the production of resin (Joseph and Stephen 1971)	Carcinogenic in the 2 year rodent bioassay (Chan et al. 1992), unknown mechanism.
	2,3,7,8-Tetrachloro-dibenzo-para-dioxin (TCDD)	Formed as a by-product in organic material synthesis and burning (Mandal 2005)	Activation of Ah receptor, oxidative stress (Knerr and Schrenk 2006)
	Nickel chloride (NiCl_2_)	Naturally occurring in soil, air, water, plants and animals. Also used as a source of nickel in chemical synthesis	Synthesis of Reactive Oxygen Species (ROS) causing oxidative stress. Epigenetic alterations (Ke et al. 2006)

The NGCs were also selected for their diverse mechanisms of carcinogenesis; 2,3,7,8-tetrachloro-dibenzo-para-dioxin (TCDD) and bis-2-ethylhexyl phthalate (DEHP) are both well-known endocrine disruptors and tumour promoters (Bock and Köhle 2005; Caldwell 2012; Casals-Casas and Desvergne 2011). Heavy metal compound nickel chloride (NiCl_2_) induces oxidative stress. The carcinogenic mechanism of methyl carbamate (MC) is less well-characterised, although MC may elicit effects via bioaccumulation (Ioannou et al. 1988).

The compounds’ relevance to human environmental exposure was a further justification (Table 1). Three of the chemicals, MMS, DEHP and MC, are also included on a recommended list of genotoxic and non-genotoxic chemicals for the assessment of the performance of new or improved genotoxicity tests (Kirkland et al. 2016).

Integrating multiple endpoints alongside genotoxicity testing was expected to provide considerably more mechanistic information to support the testing paradigm. To achieve this, the analysis of known in vivo carcinogens was performed (Table 1), with endpoints including micronucleus induction, cell-cycle alterations, cell signalling abnormalities, mitochondrial perturbations and cell morphology alterations. These endpoints cover 4 of the 6 original cancer hallmarks (Hanahan and Weinberg 2000). Results from this study have been integrated to define both genotoxic and non-genotoxic mechanisms with the future objective of developing a fully multiplexed in vitro assay for high-throughput analysis of carcinogenic potential of unknown agents.

## Materials and methods

### Chemicals

Test chemicals were purchased from Sigma-Aldrich (Haverhill, UK), with the exception of MNU (Fluorochem, Pasadena, CA, USA) and TCDD (LGC Standards, Middlesex, UK), and stored according to the manufacturer’s instructions. H_2_O_2_, MMS, MC and NiCl_2_ were dissolved/diluted in dH_2_O, whereas MNU and DEHP were dissolved/diluted in dimethyl sulfoxide (DMSO) (Thermo Fisher Scientific, Loughborough, UK).

### Cell culture

The human lymphoblastoid cell lines, TK-6 and MCL-5 (ECACC), were cultured in RPMI 1640 Medium (Life Technologies, Paisley, UK) supplemented with 10% donor horse serum and 1% l-glutamine (both Life Technologies). Hygromycin B was used to supplement MCL-5 cultures (TCDD only) to support uptake of plasmids. The cells were maintained in culture between 1  ×  10^5^ and 1  ×  10^6^ cells/ml. For all studies, cells were seeded at a density of 1 × 10^5^ cells/ml and cultured for 24 h prior to chemical treatment (37 °C, 5% CO_2_).

### Cytokinesis blocked micronucleus assay

Chromosome damage was analysed using the cytokinesis blocked micronucleus (CBMN) assay. The protocol for Metafer analysis is presented in (Seager et al. 2014). Time-points used were either 4 h treatment + 23 h recovery, or 23 h treatment + 23 h recovery. A total of 9000 binucleate cells were scored per treatment per replicate. Relative population doubling (RPD) (%) (Fellows et al. 2008; Lorge et al. 2008) was measured in parallel, with < 50% reduction in RPD relative to the vehicle control aimed for, in line with OECD requirements.

### Protein isolation and immunoblotting

To investigate p53 and phospho-p53 expression following treatment, protein isolation and immunoblotting were performed. The method followed is detailed in (Brusehafer et al. 2014).

### mRNA microarrays

mRNA microarray chip technology (Illumina, Cambridge, UK) was used to initially measure genome-wide transcriptomic changes induced by MMS, DEHP and MC at 4 and 23 h. A shortlist of genes for further qRT-PCR analysis was generated (Supplementary File 1). RNA was extracted from treated cultures using the RNeasy Mini Kit (Qiagen, Manchester, UK) following the manufacturer’s protocol. Microarray analysis was performed by Central Biotechnology Services (Cardiff University, Cardiff, UK) using an Illumina platform bead express model, with a total of 25,202 Illumina probes for known genes. Genes selected for follow-up qRT-PCR analysis were Cyclin-dependent kinase inhibitor 1A (*CDKN1A*), Choline kinase alpha (*CHKA*) and Serine/threonine protein kinase (*SGK1*).

### Gene expression analysis

qRT-PCR was completed for the aforementioned genes; the protocol is detailed in (Brusehafer et al. 2014). Primer sequences: *CDKN1A* Forward: 5′GACTCTCAGGGTCGAAAACG3′, Reverse: 5′GGATTAGGGCTTCCTCTTGG3′. *CHKA* Forward: 5′TGCAGATGAGGTCCTGTAATAAAGA3′, Reverse: 5′TTTTGGCCCAAGTGACCTCT3′. *SGK1* Forward: 5′GAACCACGGGCTCGTTTCTAT3′, Reverse: 5′GCAGGCCATACAGCATCTCAT3′. *ACTB* Forward: 5′GATGGCCACGGCTGCTTC3′, Reverse: 5′TGCCTCAGGGCAGCGGAA3′. A CFX Connect Real-time System and CFX Manager software (both BioRad, Oxford, UK) were used.

### Cell-cycle analysis

Flow cytometry was used to assess nucleated cells in G1, S and G2, where samples were processed using the In Vitro MicroFlow Micronucleus Analysis Kit (Litron Laboratories, Rochester, NY, USA), as per the manufacturer’s instructions. Samples were analysed using the BD Facs Aria Flow Cytometer (BD Biosciences, Wokingham, UK), with FacsDiva software (BD Biosciences). Appropriate gating was applied to determine the cell-cycle phase. A total of 36,000 events were analysed across 3 replicates per dose.

### Cell morphology analysis

Following treatment, cells were washed with PBS, fixed for 15 min with 4% paraformaldehyde and stained for 30 min with 2.5 µg/ml Hoechst 33,342 (Life Technologies). Brightfield and Hoechst images were acquired utilising the INCell Analyzer 2000 or 2200 (144 fields/well) (GE Healthcare, Cardiff, UK). Image analysis was performed with Matlab Version 7.12.0 (R2011a). Following this, an equal number of cell and nuclear area results were selected from a group of control replicates. These control groups were segregated depending on experimental conditions, vehicle and cell type. The smallest 20% of the population were then classified as ‘Lowest’, the next 20% as ‘Low’ and so on to classify ‘Medium’, ‘High’ and ‘Highest’ cellular/nuclear area thresholds (these being quintiles) (Supplementary File 2).

### Bioenergetics studies

The Seahorse Bioanalyzer (Agilent, Cheadle, UK) was used to measure bioenergetic flux in control and treated samples, to establish whether chemicals influenced this endpoint. Seahorse microplates (Agilent) were coated using CellTak reagent (Corning, UK). Cells pre-treated with the appropriate chemical for 4 or 23 h were transferred to coated microplates (400,000 cells/well) 1 h prior to assay commencement, with gentle centrifugation at 20×*g* to aid adhesion. Unbuffered Seahorse medium adjusted to pH 7.5 (Agilent) was used. The plate was then transferred to a non-CO_2_ incubator for 25 min prior to addition of 425 µl medium and then incubated for a further 35 min to promote equilibration. Following routine calibration of the machine, oxygen consumption rate (OCR) and extracellular acidification rate (ECAR) were measured simultaneously using the XF^e^24 Seahorse Bioanalyzer to assess basal versus drug-induced perturbations.

### ToxPi™ graphical user interface

The Toxicological Prioritization Index (ToxPi™) Graphical User Interface (GUI) is a publically available visualization tool developed at the University of North Carolina that enables the integration of multiple sources of evidence on exposure and/or safety (Reif et al. 2010, 2013). The software may be accessed via http://comptox.unc.edu/toxpi.php. Within the pie chart, the length of the “slice” radius was proportional to the magnitude of the change relative to the vehicle control. The concentration of chemical inducing a 50% reduction in RPD relative to the vehicle control, or the highest concentration administered, was used to generate fold-change values relative to the control. Slices of the pie chart were weighted according to the nature of the endpoint. Specifically, slice weightings were allocated depending on the number of endpoints measured by a single technique. All individual techniques (e.g., qRT-PCR, cell-cycle analysis) were weighted equally. Therefore, if one technique measured two or three endpoints, the sum of the weightings of these individual endpoints would be equal to the techniques with a single measured endpoint (i.e., CBMN assay and Seahorse). The square root of all values (with the exception of cell and nuclear area) was taken and scores were scaled sufficiently to enable clear “slice” visualisation for endpoint groups.

### Statistical analysis

Three biological replicates (except where indicated) were performed on separate days, with separate vials of cells/chemicals. Error bars represent standard deviation. Dose–Response Modelling with Smoothing Splines (DRSMOOTH, Mutait.org), was used to perform the statistical analysis, to identify statistically significant increases or decreases for treated samples relative to the vehicle control (Avancini et al. 2016). A mean-centering approach was used for the qRT-PCR data (Willems et al. 2008) prior to statistical analysis using DRSMOOTH. Outcomes of *p* ≤ 0.05 for two-sided tests were deemed statistically significant. For the analysis of data generated by the Seahorse Bioanalyzer, SPSS was used to perform hierarchical cluster analysis.

## Results

The study of the mechanisms by which chemical compounds in the environment may induce cancer is essential. Many in vitro-based genotoxicity tests currently only assess a single genotoxic endpoint, thus increasing the possibility of misleading predictive data. Negative results in genotoxicity and mutation-based assays for chemicals do not always equate to the chemicals being non-carcinogens, considering that a subset of carcinogens are non-genotoxic. Therefore, it is emerging that the use of more sophisticated, multiple-endpoint in vitro approaches will better inform safety assessment while minimizing laboratory animal use. Multiple endpoints allow a holistic overview of chemicals’ effects on cells, leading to greater mechanistic understanding for both genotoxic and non-genotoxic carcinogens. Here, a novel integrated test strategy was developed using a variety of carcinogenicity-associated endpoints.

### The GCs caused genotoxicity

**Fig. 1:**
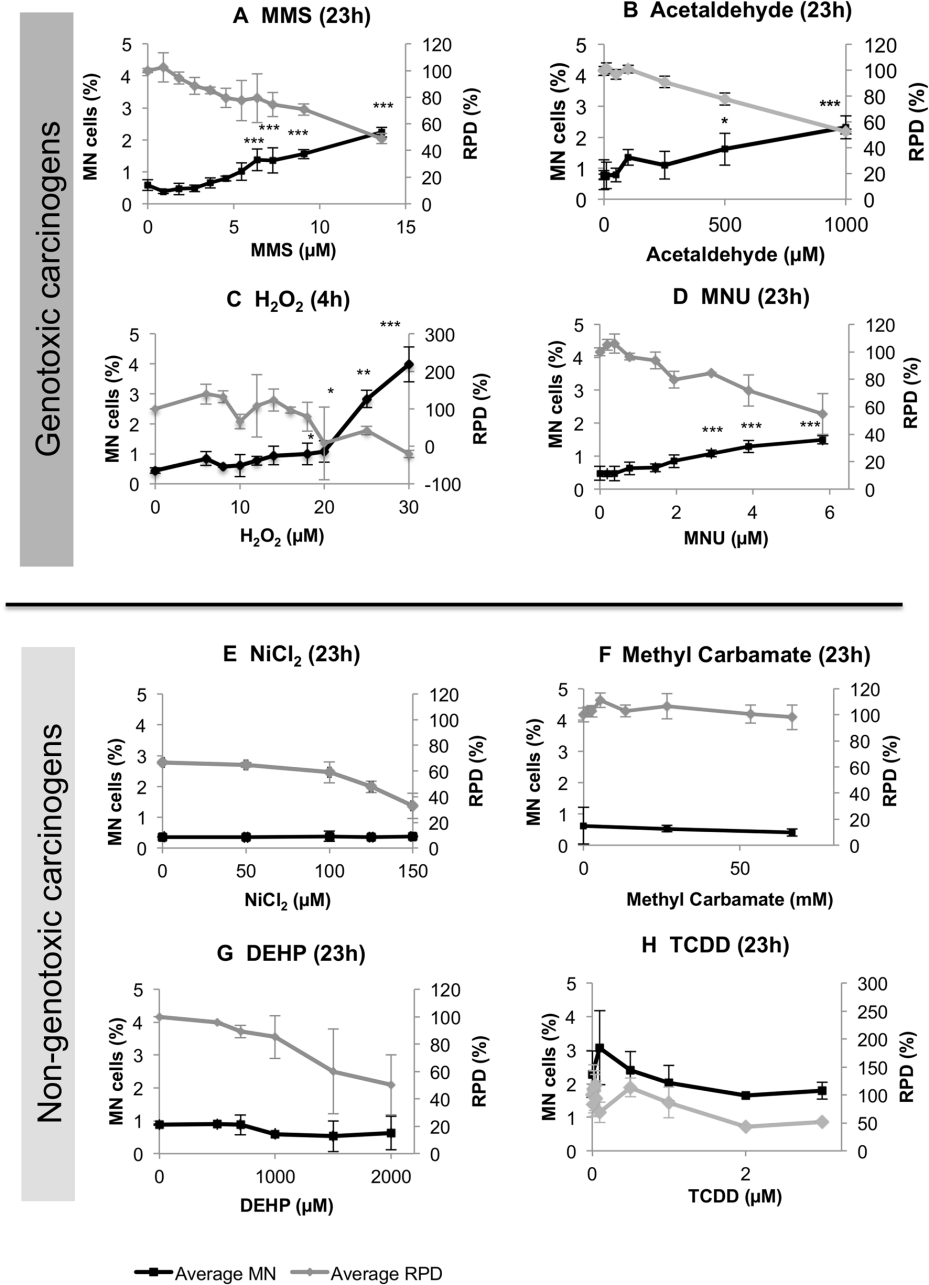
The CBMN assay (4 or 23 h exposure + 23 h recovery) was used to determine whether test compounds were genotoxic. Micronucleated cells (%) (black lines) and Relative Population Doubling (RPD) (%) (grey lines) data are displayed for eight chemicals (*n* = 3 for **a**–**d**, *n* = 2 for **e**–**h**). Data for NGCs are presented in duplicate due to two replicates being sufficient to confirm the lack of MN-induction for these chemicals. Statistically significant changes in percentage cells relative to the vehicle control are denoted by *, where *p ≤ 0.05, **p ≤ 0.01 and ***p ≤ 0.001. For RPD, a Beckman Coulter Counter was used to count cells prior to dosing and following the recovery period

Genotoxicity induction was measured using a high-powered CBMN assay (Fig. 1). Cells were treated with test chemical for 4 h (+ 23 h recovery) initially to identify no-observed effect levels (NOELs) and lowest observed effect levels (LOELs) for micronucleus (MN) dose–responses. If no significant increases in genotoxicity (i.e., LOELs) were observed after 4 h + 23 h, 23 h treatment was performed (+ 23 h recovery). H_2_O_2_ was the only positive chemical after 4 h (Fig. 1c). The three other GCs, acetaldehyde, MMS and MNU, did not produce significant changes in genotoxicity after 4 h (data not shown). At 23 h, however, all of these chemicals caused MN induction at concentrations of ≥ 500, 6.4 and 2.9 µM, respectively (Fig. 1).

No NGCs tested, DEHP, MC, NiCl_2_, and TCDD, induced significant MN increases at any test concentrations after 4 or 23 h. As TCDD is known to induce enzymes such as the Cytochrome P450 s (Hukkanen et al. 2000), it was tested using the metabolically competent MCL-5 cell line (Fig. 1h); here, no significant increases in MN frequency were observed (*p* > 0.05). MC was the only chemical not to approach 50% cytotoxicity. Dose selection for MC was performed based on literature, hence the maximum concentration exceeded the recommended 10 mM (Kim et al. 2005; Kwon et al. 2007; Mitchell et al. 1997).

### p53 and phospho-p53 increased in response to all genotoxic and one non-genotoxic chemical

**Fig. 2:**
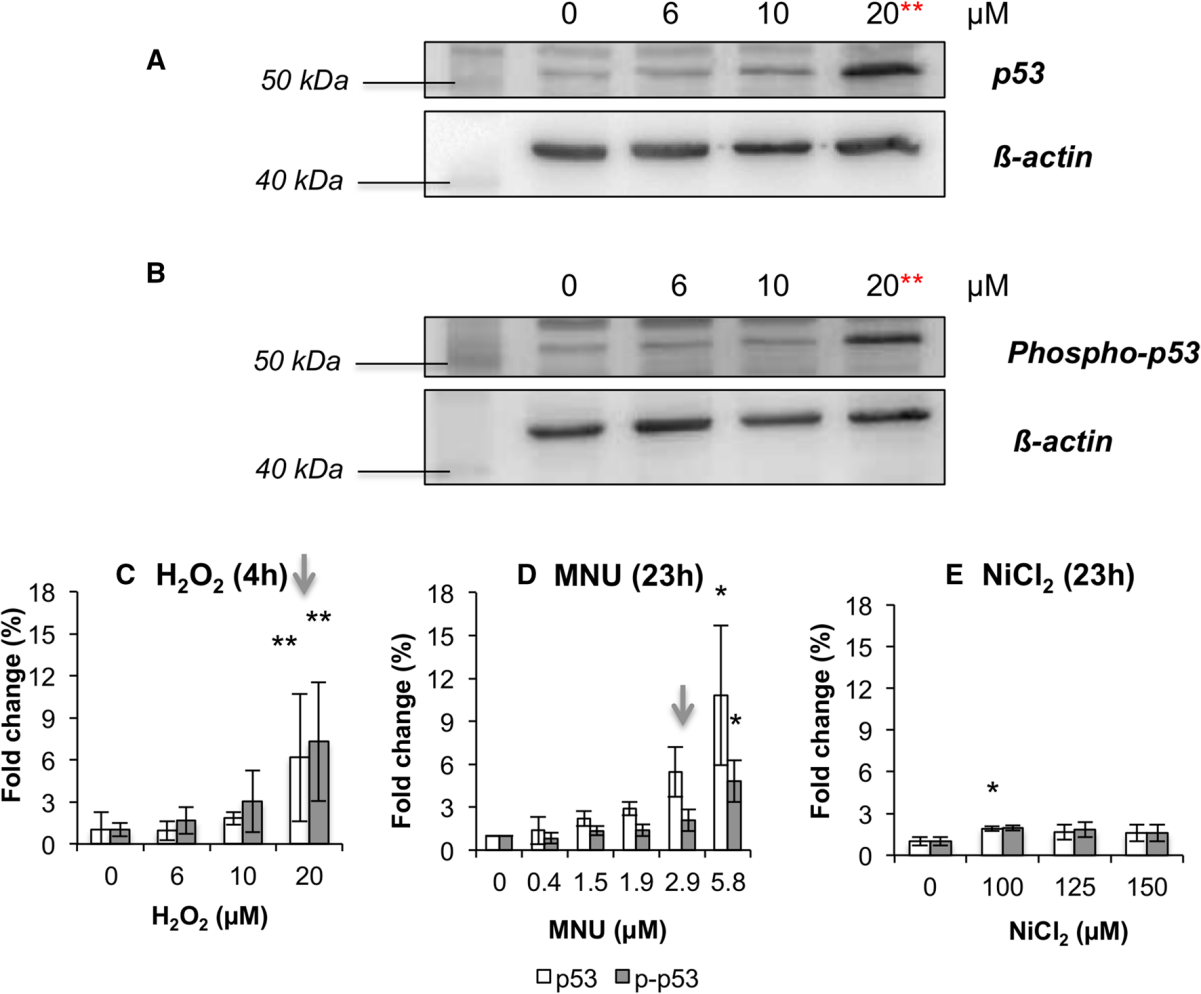
p53 and phospho-p53 expression as determined by Western blotting for 4 or 23 h exposure. **a**, **b** Representative examples of blot images for p53, phospho-p53 (Both 53 kDa) and ß-actin (45 kDa). These examples were from H_2_O_2_ treatment. **c**–**e** Densitometry graphs are presented for chemicals that caused significantly altered expression of p53 and/or phospho-p53 (*n* = 3). Statistically significant changes in fold change expression are denoted by red *, where *p ≤ 0.05, **p ≤ 0.01 and ***p ≤ 0.001. Arrows above specific concentrations correspond to the MN LOEL, or concentration nearest to the LOEL, for the carcinogens (Fig. 1), where applicable (colour figure online)

p53 is an important node in the DNA Damage Response (DDR) with a central role in carcinogenesis (Banin et al. 1998). p53 and phospho-p53 protein levels were assessed using Western blotting (immunoblotting) and the relevant bands on images quantified using densitometry (Fig. 2). For GCs, MNU and H_2_O_2_ induced p53 and phospho-p53 at concentrations above their respective genotoxicity LOELs (Fig. 2c, d). MMS and acetaldehyde also increased p53 and phospho-p53, although these changes were non-significant (Supplementary File 3). As Western blotting is only semi-quantitative, it is plausible that such changes indicate true biological effects, despite lacking significance. Treatment with the NGC NiCl_2_ also elevated p53 levels, despite this chemical not inducing genotoxicity (Fig. 1). NiCl_2_, however, did not cause a dose-dependent increase, with only 100 µM increasing p53 abundance. Interestingly, NiCl_2_ was the only chemical tested to significantly induce reactive oxygen species (ROS), (Supplementary File 4), possibly explaining this unusual result. DEHP, MC and TCDD did not cause significant changes in p53 or phospho-p53 protein abundance (Supplementary File 3).

### Carcinogens altered p21, CHKA and SGK1 mRNA expression

Whole-genome RNA microarrays were used to determine a small panel of target genes altered by DEHP, MC and MMS for further, more detailed gene expression studies by qRT-PCR. Following microarray analysis, three “carcinogenesis biomarker” genes were taken forward for further investigation: *CDKN1A*, *CHKA* and *SGK1* (highlighted in Supplementary File 1). *CDKN1A* encodes p21^Cip/Waf^; due to its relevance to cancer, this gene was selected independently of the microarray data. The other two genes, *CHKA* and *SGK1*, were selected based on the criteria outlined in Supplementary File 1, Tab 2. *CHKA* is known to be over-expressed in human tumours (de Molina et al. 2002), while *SGK1* regulates survival and growth in colorectal cancers (Lang et al. 2010).

**Fig. 3:**
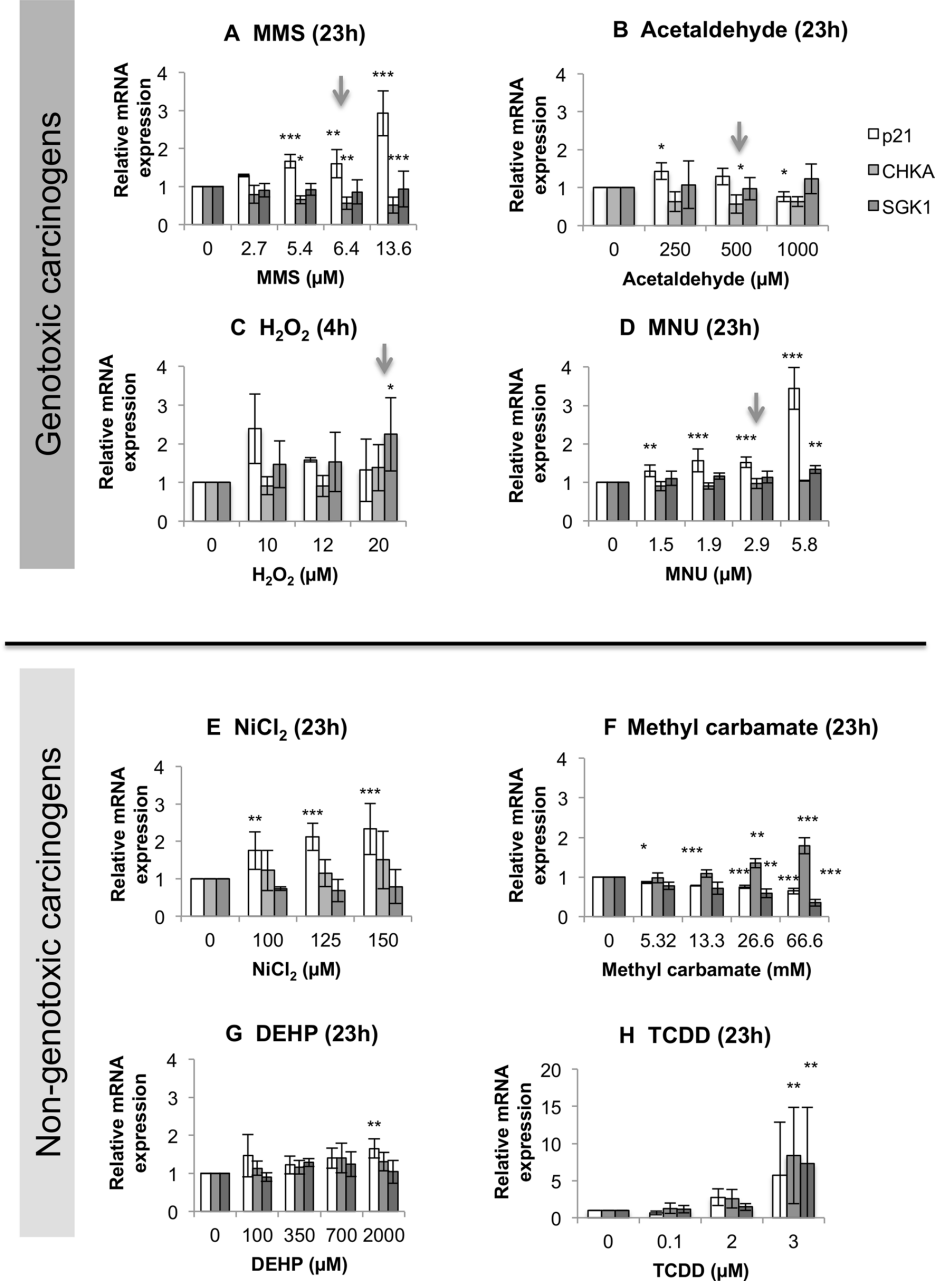
Relative expression of mRNA of the *CDKN1A*, *CHKA* and *SGK1* genes as determined by qRT-PCR (*n* ≥ 3) for 4 or 23 h exposure. Statistically significant changes in fold change gene expression relative to the vehicle control are denoted by *, where *p ≤ 0.05, **p ≤ 0.01 and ***p ≤ 0.001. Arrows above selected concentrations correspond to the MN LOEL, or concentration nearest to the LOEL, for carcinogens, where applicable

Two GCs, MMS and MNU, induced clear dose-dependent increases in mRNA transcribed from the p21 gene (Fig. 3c, d). Acetaldehyde stimulated a biphasic dose-response, with an increase at 250 µM, followed by a decrease at 1000 µM. Indeed, all GCs increased p21 expression, despite levels of p21 induced by H_2_O_2_ lacking significance. It was noted that acetaldehyde, MMS and MNU produced significant increases in expression of p21-encoding mRNA at concentrations below the MN LOELs (Fig. 1), a phenomenon not observed with Western blotting for p53 activation. MMS and acetaldehyde both significantly altered *CHKA* gene expression relative to the control (Fig. 3, Supplementary File 3). H_2_O_2_ and MNU significantly altered *SGK1* expression at the highest test concentrations (Fig. 3). With the exception of MMS, all GCs increased *SGK1* mRNA expression above control levels (Fig. 3, Supplementary File 3).

NGCs also demonstrated a capacity to alter gene expression (Fig. 3e–h). Three NGCs significantly altered p21 mRNA expression: DEHP, NiCl_2_ and MC (Fig. 3). NiCl_2_ produced a clear dose-dependent increase in p21 mRNA (Fig. 3e). Interestingly, MC significantly reduced both p21 and *SGK1* mRNA levels, in contrast to the GCs that increased their expression. Two NGCs, MC and TCDD, significantly altered *CHKA* expression (Fig. 3). However, all NGCs increased *CHKA* mRNA expression (Supplementary File 3). MC and TCDD were also the only NGCs to significantly alter *SGK1* levels. In summary, all eight chemicals caused statistically significant dysregulation of at least one of the genes tested.

### Four test chemicals induced arrest at G2 phase of the cell-cycle

**Fig. 4:**
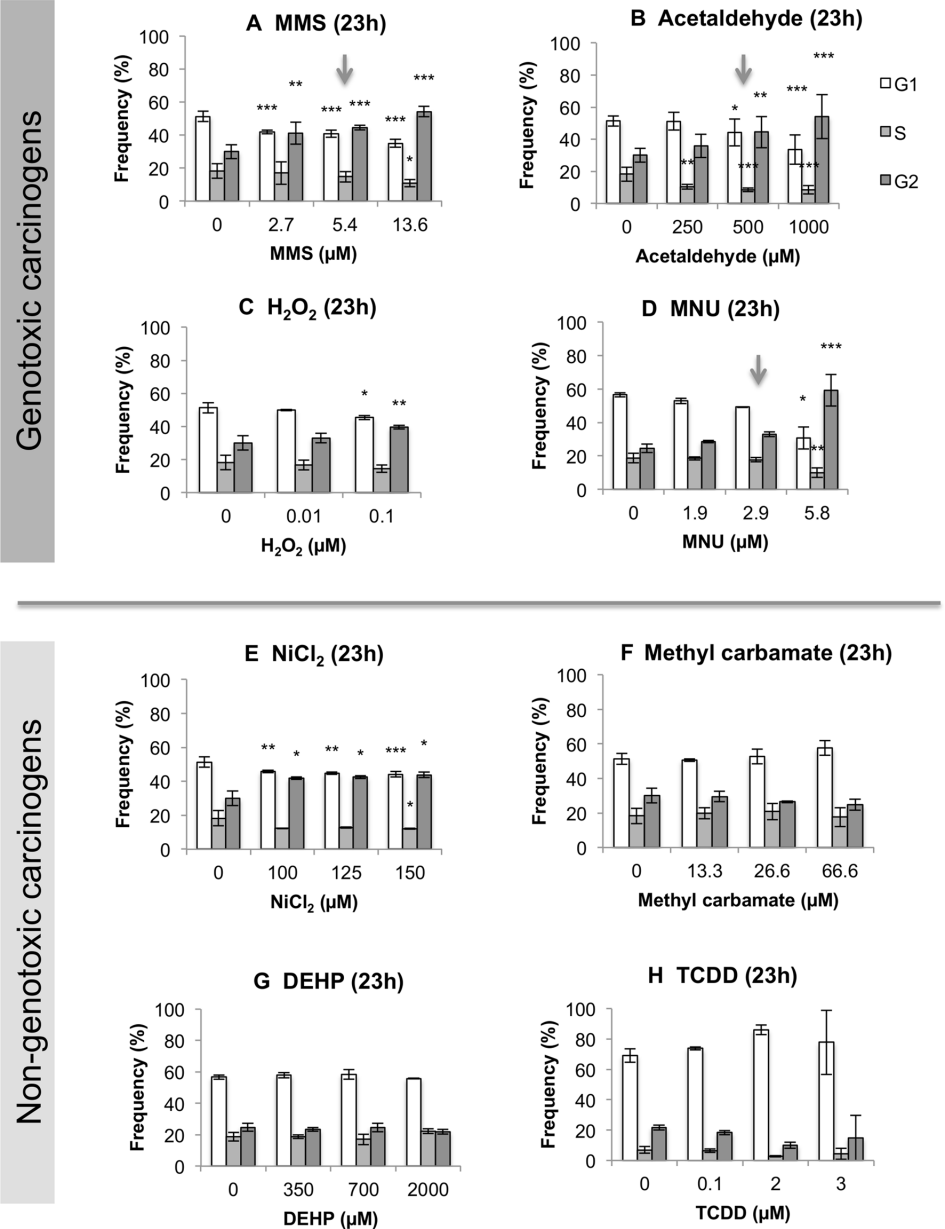
Cell cycle analysis was performed using flow cytometry for samples treated for 23 h (*n* = 3). A historical vehicle (either H_2_O or DMSO) control was used for all chemicals. Statistically significant changes in percentage cells relative to the vehicle control are denoted by *, where *p ≤ 0.05, **p ≤ 0.01 and ***p ≤ 0.001. Arrows above selected concentrations correspond to the MN LOEL for carcinogens, where applicable

As the cell-cycle is a crucial link to the cancer hallmark of uncontrolled proliferation, the distribution of nucleated cells in G1, S and G2 cell-cycle phases immediately following 4 or 23 h treatments was measured using flow cytometry (Fig. 4). All GCs stimulated statistically significant and, in the case of MMS and acetaldehyde, dose-dependent, increases in cells in the G2 phase after 23 h, indicating G2 arrest (Fig. 4a–d). These G2 increases were accompanied by statistically significant reductions in the two other cell-cycle categories, G1 and S phase. The GC H_2_O_2_ did not induce any statistically significant changes in the cell-cycle distribution at 4 h (data not shown); therefore, a 23 h exposure with lower H_2_O_2_ concentrations was completed (Fig. 4a). Lower concentrations were used for 23 h than 4 h, to prevent reductions in RPD exceeding 50%. Similarly to three of the GCs, the NGC NiCl_2_ (Fig. 4e) caused G2 cell-cycle arrest, with this being observed at all tested concentrations (i.e., ≥ 100 µM). No significant alterations in cell-cycle were observed for the remaining NGCs (Fig. 4f–h).

### The majority of chemicals caused cell and nuclear morphological changes

Cell morphological changes have previously been associated with metastasis and invasion (Grünert et al. 2003; Tsai and Yang 2013) and are the basis of CTAs. Metastasis is closely associated with cancer mortality in humans and invasion links to the epithelial to mesenchymal transition (EMT). Therefore, cell morphology may provide a powerful early indicator of carcinogenesis-associated alterations.

**Fig. 5:**
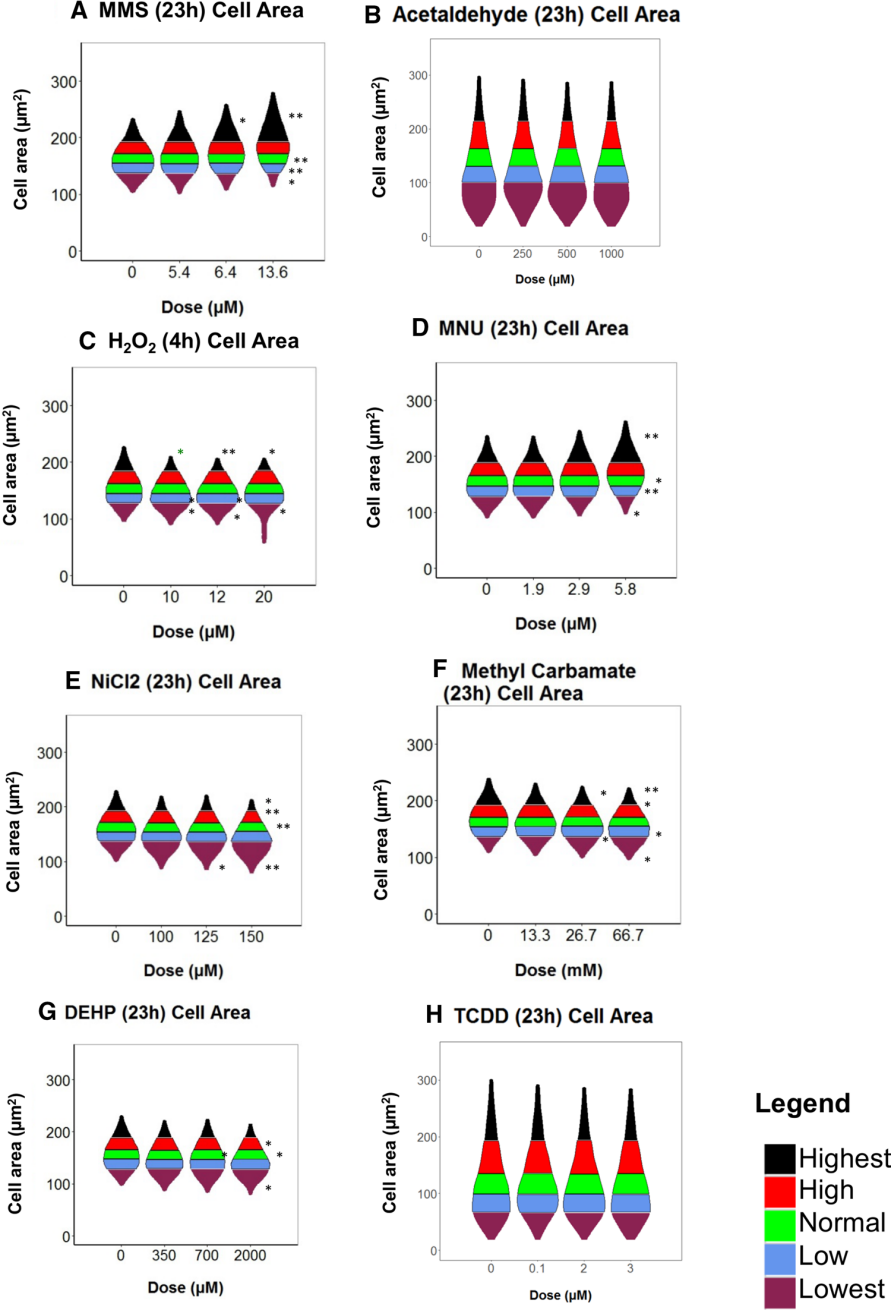
Violin plots displaying cell area changes within data obtained via the INCell Analyzer, followed by Matlab-based image analysis. The frequency of cells (%) in each quintile category is plotted. The cell area ranges represented by the quintiles are included in Supplementary File 2. Alterations of the percentage of cells/nuclei within these set ranges were compared following treatment with carcinogens. Statistically significant changes in percentage cells relative to the vehicle control are denoted by *, where *p ≤ 0.05, **p ≤ 0.01 and ***p ≤ 0.001. These are summarised in Supplementary File 3. No recovery time (i.e., 0 h) was allowed following the treatment period, due to data collection at 8 and 23 h recoveries consistently demonstrating reduced effects on morphology compared to 0 h. Other morphology endpoints, such as cell and nuclear perimeter, solidity and form factor were also determined using the script-based analysis method, increasing confidence in the cell and nuclear area changes observed (colour figure online)

**Fig. 6:**
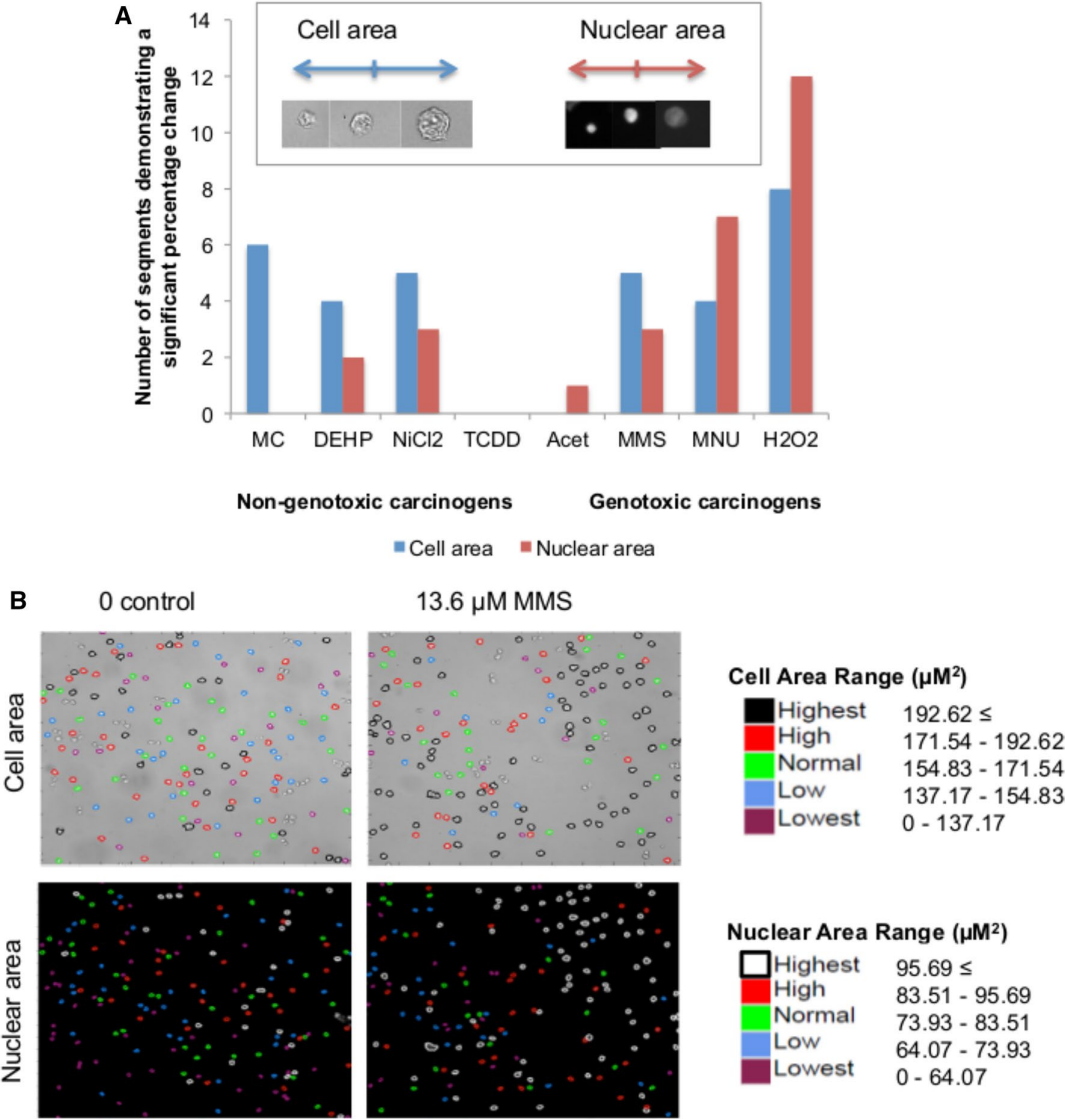
a Graphical summary of the total number of quintiles producing statistically significant changes relative to the untreated control for the two different morphological endpoints: cell area (Fig. 5) and nuclear area (Supplementary File 5). Inset: Example images of randomly selected “small”, “medium” and “large” cell and nuclear images captured using the INCell Analyzer 2000. **b** Colour-coded cell and nuclear perimeters overlaid on randomly selected raw images obtained via the INCell Analyzer, to illustrate an increase in cell (black outlines) and nuclear (white outlines) area (µm^2^) following 13.6 µM MMS treatment (colour figure online)

Three out of four GCs significantly altered cell area (Fig. 5) relative to the vehicle control. The NGCs generally gave a greater response for cell area than for nuclear area, which contrasted with some of the GC data. With the exception of TCDD, the NGCs caused a significant reduction in cell area, each significantly increasing the “Lowest”/”Smallest” cell category (Fig. 5e–g). The greatest cell area decrease was observed with NiCl_2_, which caused the percentage of cells < 137 µm^2^ to decrease by 23% (Fig. 5e). TCDD (Figs. 5, 6h) was the only chemical that did not alter cell or nuclear area.

For nuclear area, a greater level of significance was generally observed for the GCs than for cell area (Figs. 5, 6, Supplementary File 5). For example, MNU produced a highly significant (*p* < 0.0002) increase in nuclei of > 90.1 µm^2^ from 19 to 38%. H_2_O_2_ caused the “Smallest” range of the nuclei (Fig. 6a) (< 95 µm^2^) to increase more than threefold, from 20 to 64%. In addition, acetaldehyde did not have any significant effect on cell area whilst a significant, 5% decrease of “Small” sized nuclei was observed. The extent of statistical significance for the two morphology endpoints is summarised in Fig. 6.

### Bioenergetics analysis revealed trends for carcinogens

**Fig. 7:**
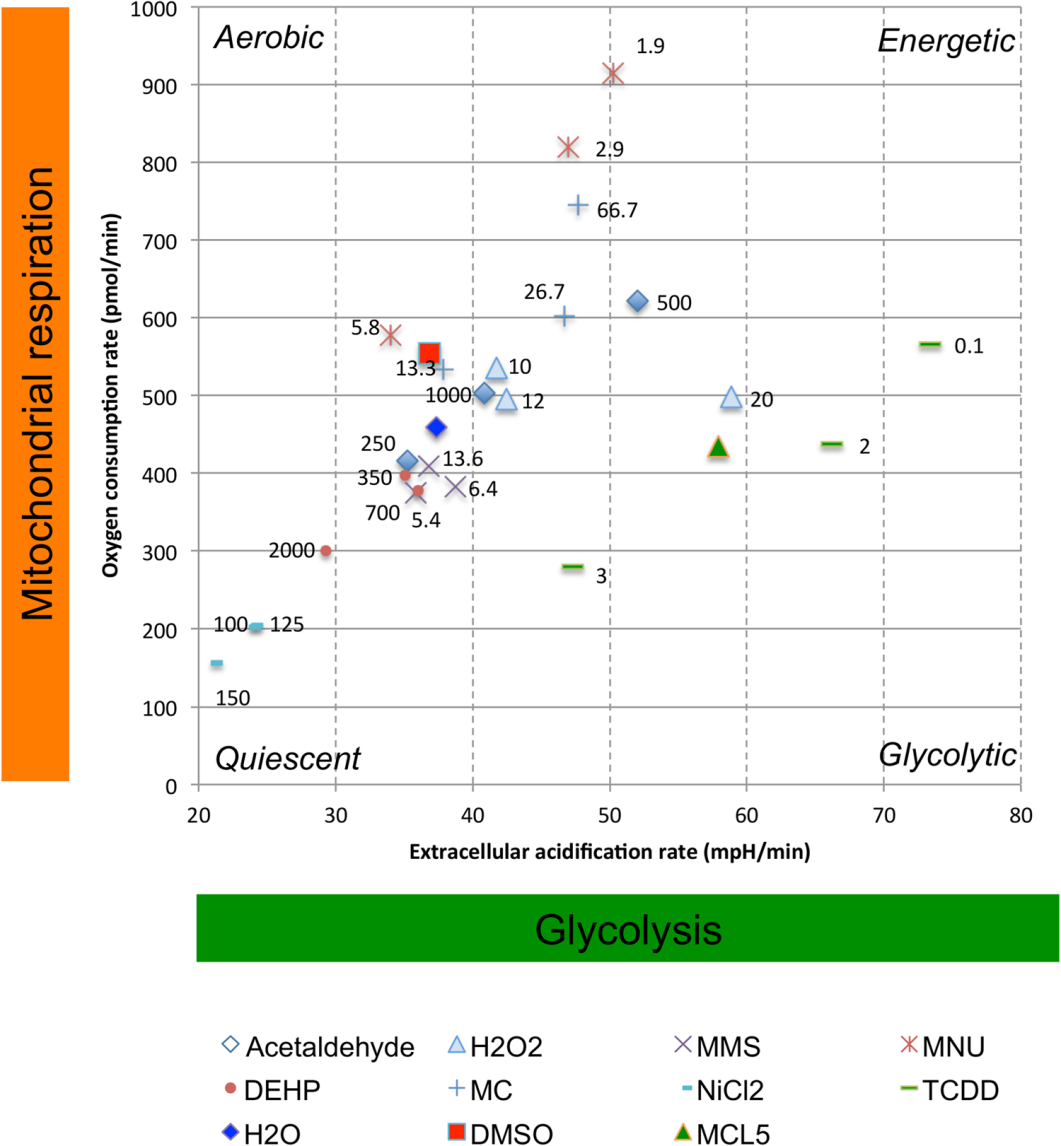
Bioenergetics analysis of control and treated cells using the Seahorse XF^e^24 Bioanalyzer (*n* ≥ 3) to establish whether chemicals induced a “stressed” phenotype. OCR is plotted against ECAR for basal cellular metabolic rates. Historical vehicle controls (H_2_O, DMSO) are included and blue points correspond to H_2_O chemicals, whereas red points correspond to DMSO chemicals. Green points correspond to TCDD, which was analysed in a different cell line (MCL-5). Cluster analysis was performed on the data. Four bioenergetic states were included [Quiescent, aerobic (oxidative phosphorylation), glycolytic and energetic (glycolytic + oxidative phosphorylation)] that represent the energy phenotype (colour figure online)

Mitochondrial and glycolytic flux were measured using the Seahorse XF^e^24 Analyzer (Fig. 7) to determine whether carcinogens influenced cellular bioenergetic profiles. Figure 7 models the shift from vehicle controls towards “stressed” phenotypes following chemical treatment (Robinson et al. 2012). Although no changes were significant, general trends were apparent. MNU, MC and acetaldehyde induced a shift towards an “energetic” phenotype. NiCl_2_, DEHP and MMS shifted cells towards quiescence, reducing both OCR and ECAR. NiCl_2_ reduced OCR by almost threefold at 150 µM, from 459 pmol/min to 156.8 pmol/min, while simultaneously reducing ECAR by 1.8-fold. Interestingly, NiCl_2_ also elevated ROS concentrations, as mentioned previously (Supplementary File 5). H_2_O_2_ caused a more glycolytic phenotype, whereas TCDD demonstrated contrasting trends at different concentrations.

### Endpoints were summarized using the ToxPi GUI

**Fig. 8:**
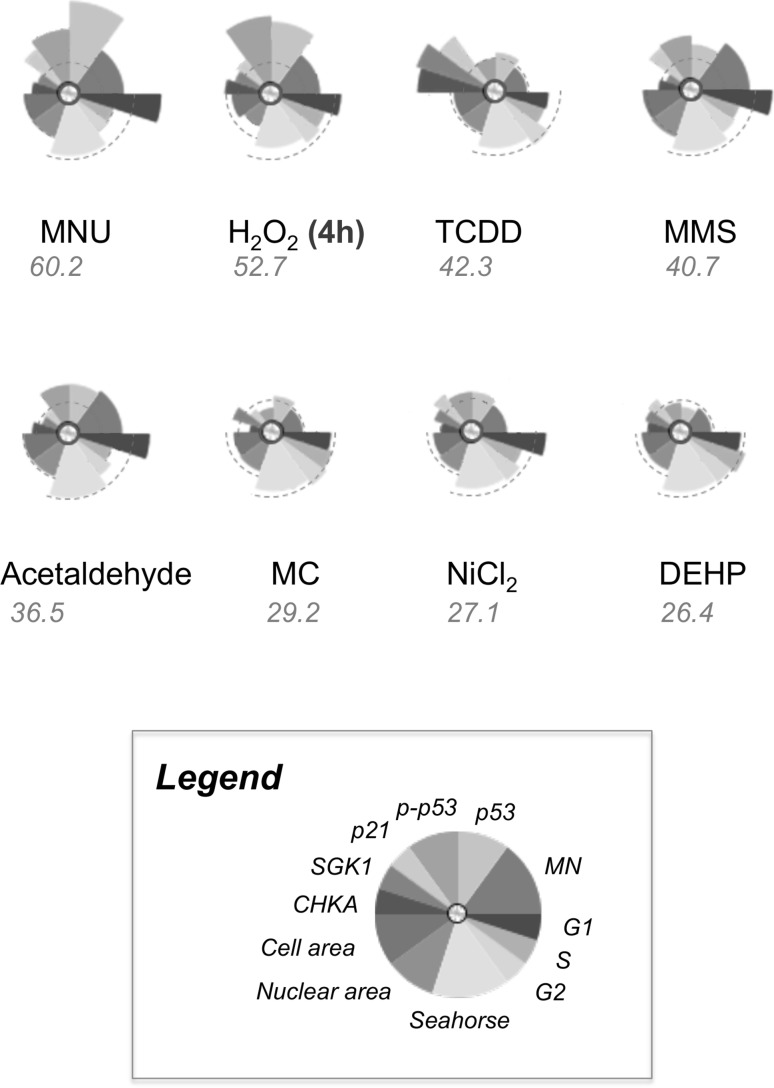
Outputs from the Toxicological Prioritization Index (ToxPi) GUI summarising the fold changes for the endpoints at the 50% RPD-inducing concentration. Broken lines indicate the position of “one-fold” for the relevant endpoints. Carcinogens were ranked according to their potency scores, from highest to lowest. Fold changes were square rooted, transformed and weighted as appropriate (i.e., 3 for each of “MN” and “Seahorse”, 1.5 for each of “cell area”, “nuclear area”, “p53″ and “phospho-p53”, and 1 for each of the remaining endpoints), and values < onefold were inverted to give values > onefold (for down-regulation). The sum all values for all endpoints was taken for each chemical, generating a final overall score. Data were scaled to provide three separate groups of “onefold” magnitude, due to notable variation in the magnitude of changes for different endpoints and, therefore, to ensure clear visualisation of all endpoint “slices” (colour figure online)

To visualise trends for different endpoints and to rank the chemicals in terms of their toxicological impact, ToxPi GUI was used to generate a diagrammatical representation for each chemical (Fig. 8). Composite scores for all endpoints were generated for each chemical, in an attempt to predict their rank order in terms of carcinogenic effects.

In terms of the ToxPi profiles, the GCs produced broadly similar distributions, altering similar endpoints, in particular p53, phospho-p53, cell-cycle distribution, cell and nuclear area, and MN frequency. Within the five highest-ranking scores, four of these were GCs, with scores ranging from 60.2 for MNU to 36.5 for acetaldehyde. Meanwhile, H_2_O_2_ produced a score of 52.7 and MMS, 40.7. It is important to note that H_2_O_2_ was the only chemical where endpoints were measured at 4 h, rendering it the most potent compound overall despite not achieving the greatest score.

NGCs generally produced the lowest scores, with three ranking 6th–8th, as follows: MC (29.2), NiCl_2_ (27.1) and DEHP (26.4). This complemented the fact that NGCs altered fewer carcinogenicity endpoints than GCs. The ToxPi profiles displayed noticeable similarities between these three chemicals, despite p21, p53 and cell-cycle arrest being induced by NiCl_2_ only. TCDD, however, elicited a greater effect than other NGCs, producing the third highest score (42.3). This high rank was almost entirely due to the large gene expression increases induced by TCDD, as this chemical did not alter any other endpoints. As a result, TCDD’s ToxPi profile indicates a somewhat unique response compared to other chemicals, differing from that of either carcinogen group.

Furthermore, the ToxPi profiles and accompanying rank order demonstrated potential for read-across between carcinogen classes, indicating some separation between GCs and NGCs, with GCs generally inducing greater responses for these endpoints.

## Discussion

The accurate prediction of a novel chemical’s carcinogenic potential in humans is crucial if cancer prevention is to be a possibility. Analysis of phenotypic changes of human cells in response to carcinogens is essential for fully understanding human oncogenesis. Holistic testing of carcinogens offers many advantages over the testing of isolated endpoints (Benigni 2014; Bourcier et al. 2015; Breheny et al. 2011; McKim and James 2010), ranging from improved predictivity to reduced time and financial costs (Kirsch-Volders et al. 1997; Stankowski et al. 2015). The use of in vitro testing approaches and chemical mode-of-action identification is currently favoured (Adeleye et al. 2015; EPA 2005; Thybaud et al. 2007). Indeed, many mechanism-centric in vitro tests using “next generation” approaches for identifying carcinogens have been developed (Caiment et al. 2013; Gusenleitner et al. 2014; Herwig et al. 2016; Tilton et al. 2015), with these linking to cancer hallmarks or toxicity prioritisation (Dix et al. 2007; Kleinstreuer et al. 2012; Smith et al. 2016).

This study’s objective was to further develop such approaches, determining whether the carcinogenic potential of known in vivo carcinogens could be successfully identified via an in vitro, multi-endpoint test system, with particular interest in identifying NGCs. Ten molecular and cellular “surrogate” carcinogenicity endpoints reflecting the “Hallmarks of Cancer” (Hanahan and Weinberg 2011) were selected to test eight carcinogens.

### Multi-endpoint analysis provided more informative risk assessment

A flow diagram was created to summarise the relationships between the endpoints, or “adverse outcomes” (Supplementary File 6), based on the data. Generally, similar trends for the GCs were apparent for p53, p21 and the cell-cycle, reflecting the outcomes of studies such as (Lukas et al. 2004). Cell morphology, however, indicated some diversity in trends for GCs: MMS and MNU increased cell and nuclear area, in agreement with relative cellular size at G2 phase (Figs. 5, 6). In contrast, H_2_O_2_ markedly reduced cell and nuclear area (Figs. 5, 6), possibly linking to its shorter exposure duration (4 h). However, this also reflects some NGC trends, perhaps suggesting a ROS-centric mechanism (Stannard et al. 2016). It was hypothesised that the mammalian target of rapamycin (mTOR) may orchestrate cell morphology alterations (Fumarola et al. 2005; Llanos et al. 2016; Pincus and Theriot 2007). Indeed, we have noted that mTOR-inhibitor rapamycin reduced cell and nuclear area, indicating effects similar to some test carcinogens (Supplementary File 7). In general, NGCs induced fewer significant effects than GCs, with  these mainly involving gene expression and cell morphology alterations (Figs. 3, 5, 6, Supplementary File 6). No significant effects were observed for bioenergetics, which may be unsurprising when using low-doses; however, this endpoint remains valuable for carcinogenicity testing. The use of holistic endpoints could be considered synonymous with “key events” of the Adverse Outcome Pathway (AOP) concept. However, the present approach avoids the limitations of focusing on a single pathway, as a combination of both molecular- and cellular-level changes are considered.

Data for the multiple endpoints could, with further optimisation, be multiplexed within a single, high-content system, such as the INCell Analyzer. For example, MN and cell-cycle data can already be collected simultaneously via this approach. Furthermore, while endpoints were selected based on their relationship to the “Hallmarks of Cancer”, one of the major original hallmarks, resistance to apoptosis, was not included. This is due to the low concentrations of chemical used inducing only minimal levels of apoptosis, meaning that resistance to apoptosis would be difficult to measure effectively.

Another important aspect of validation relates to “non-carcinogens”, as it is necessary to ensure that such chemicals deliver negative results. Extensive validation of this class is beyond the scope of the present study. However, the vehicles used, H_2_O and DMSO, are non-carcinogens and did not adversely alter the endpoints tested. The lack of effect for these chemicals provided support for the assay’s specificity.

### The CBMN assay exhibited limited sensitivity for detecting carcinogenic outcomes

Importantly, for the GCs, alterations in other, non-MN endpoints (Figs. 3, 4) often occurred at concentrations lower than the LOEL for MN frequency. This suggests that other, non-genotoxicity endpoints offer greater sensitivity for GC detection than the CBMN assay. This may be due to the efficient removal of potentially clastogenic DNA lesions via DNA repair mechanisms at low doses; should such lesions remain unrepaired, these may also not necessarily cause the “late” cellular events that are MN (Fenech 1997). These protective factors reduce the frequency of observed clastogenic events (e.g., MN), and so the full DNA damage profile induced by the chemical may not be evident. The fact that the CBMN assay is not designed to detect NGCs further supports the use of multi-endpoint testing, particularly considering NGCs’ diverse mechanisms.

Importantly, all chemicals caused at least one statistically significant change in the endpoints tested; this again supports the use of multiple endpoint tests, as these may reduce the probability of “missing” biological impacts of carcinogens. No chemicals exhibited adverse effects at all concentrations tested for all endpoints, with low concentrations, unsurprisingly, being less likely to induce an effect.

### Discrete categories of carcinogens may be irrelevant: NiCl_2_ exhibited GC-like effects

While this study has provided mechanistic insights for individual carcinogens (Supplementary File 6), the overall, integrated results for chemicals were also informative. The resulting scores (Fig. 8), when ranked from highest to lowest, indicated a general separation between GCs and NGCs, with four of the five highest scores belonging to GCs. However, despite GCs and NGCs potentially affecting different endpoints, the incomplete separation between these groups suggested that carcinogens should be analysed on a case-by-case basis. Therefore, this study proves that dividing carcinogens into discrete categories such as “genotoxic” and “non-genotoxic” may be an oversimplification, a case in point being NiCl_2_. NiCl_2_ conferred several effects that overlapped with those of GCs, such as p53 activation and G2 cell-cycle arrest, despite not being observed to induce genotoxicity in this study (Fig. 1) or in some other studies (Biggart and Costa 1986; Chakrabarti et al. 2001). Therefore, NiCl_2_ may not be a true NGC, as was previously believed, and its genotoxicity may be dependent on its exposure time (Stannard et al. 2016). It is, therefore, apparent that different groups of carcinogens have a unique in vitro “fingerprint” or “signature” for carcinogenicity. This could be termed the “Integrated Signature of Carcinogenicity” (ISC), representing the overall, multiple-endpoint response of cells in vitro to any test chemical (Fig. 8). With further validation, it is possible that a “cut-off” ISC value could be identified, enabling GCs to be distinguished from NGCs.

### In vitro and in vivo rankings were broadly aligned

Relating rank order, or ISCs, to in vivo carcinogenicity data may be informative, particularly as such an approach may replace the two-year rodent carcinogenicity bioassay for non-pharmaceuticals, impacting on the chemical industry. TD_50_ data for the chemicals (Gold database) are listed below:

TCDD: 0.000023 mg/kg/day; rat

MNU: 0.0927 mg/kg/day; rat

MMS: 32 mg/kg/day; mouse

MC: 56 mg/kg/day; rat

Acetaldehyde: 153 mg/kg/day; rat

DEHP: 716 mg/kg/day; rat

H_2_O_2_: 7,540 mg/kg/day; mouse

NiCl_2_: Data unavailable

The in vitro and in vivo data indicated broad agreement: three of the four most potent in vivo carcinogens, based on these chemicals’ TD_50_ doses, corresponded with the ToxPi rankings for the 50% RPD concentrations, despite a slightly different ranking order; however, H_2_O_2_ appears to be considerably less potent in vivo, being ranked last. Interestingly, H_2_O_2_ was the most potent chemical in vitro, being the only chemical to induce genotoxicity after 4 h while producing the second highest ToxPi score. This difference could be explained by the greater antioxidant capacity in vivo (Niki 2010) compared to in vitro systems, which are known to be hyperoxic and devoid of protective antioxidants. Another explanation may relate to the in vivo method of exposure being via the animals’ water, contributing to losses of unstable H_2_O_2_ to, for example, digestive system microbiota. The highest-ranking in vivo carcinogen was TCDD, whereas in vitro, GCs ranked higher.

## Conclusions

The present study has established that a multiple-endpoint approach is a more comprehensive means of assessing carcinogenicity of environmental carcinogens in vitro than traditional, single-endpoint tests. Crucially, this novel testing strategy will provide a means of in vitro NGC detection. Advantages of our approach include use of low-doses, automated technology and genetically stable human cells. Such a test could eventually provide sufficient information to replace the two-year rodent carcinogenicity assay for non-pharmaceuticals, reducing animal use in carcinogenicity assessment. Further data for other chemicals and cell models, such as liver, are now required to verify these observations.

## Electronic supplementary material

Below is the link to the electronic supplementary material.

**Supplementary File 1** Data summary for the RNA microarray analysis, including fold-change and p-values. The selected genes for follow-up qRT-PCR, *CDKN1A*, *CHKA* and *SGK1* are highlighted in colour in the TargetID column (XLSX 168 kb)

**Supplementary File 2** Table summarizing the threshold values for the cell morphology analysis based on images captured by the INCell Analyzer 2000. Quintile ranges are displayed for both cell area (µm^2^) and nuclear area (µm^2^), with these set based on the vehicle control data. Separate thresholds were calculated for different solvents, exposure durations and the cell line used (XLSX 10 kb)

**Supplementary File 3** Table summarising the fold-change results of cell signaling studies in response to chemical carcinogens. The concentration inducing the maximum significant effect is displayed. Arrows indicate the direction of change relative to the control and are only included for statistically significant results. If no significant change was observed, the lowest concentration inducing a quantitative change is displayed (TIFF 1521 kb)

**Supplementary File 4** Reactive oxygen species levels were studied using a standard DCFDA methodology. Readings of treated cells were taken at 4 h, 6 h and 24 h. H_2_O_2_ and NiCl_2_ produced significant increases in ROS evolution (TIFF 138 kb)

**Supplementary File 5** Violin plots displaying nuclear area changes from data obtained via the INCell Analyzer, followed by Matlab-based image analysis. The frequency of cells (%) in each quintile category is plotted. Statistically significant changes in percentage cells relative to the vehicle control are denoted by *, where * = p ≤ 0.05, ** = p ≤ 0.01 and *** is p ≤ 0.001 (TIFF 178 kb)

**Supplementary File 6** Flow diagram illustrating the holistic nature of the “adverse outcomes” studied, based on the general results. Blue outlines indicate a series of events primarily associated with GCs, while green outlines indicate NGC-associated events. Orange indicates events that may be involved in either carcinogenic mechanism. Extracts from figures are included for illustrative purposes. An alternative, bar chart-based method of presenting the cell morphology data is indicated(TIFF 100 kb)

**Supplementary File 7** Cell and nuclear data obtained using the INCell Analyzer 2000 for mTORC1 inhibitor, rapamycin, in TK6 cells (n = 2). A. Rapamycin (23 h + 0 h treatment) induced a reduction in cell area (n = 2), in agreement with previous observations (Fingar and Blenis, 2004). B. A similar reduction in nuclear area was observed in response to 23 h treatment with non-genotoxic carcinogen methyl carbamate. Asterisks represent p < 0.05. The concentrations included those inducing up to 50% cytotoxicity to limit non-chemical specific secondary toxicity effects. C. Colour-coded cell and nuclear perimeters overlaid on randomly selected raw images obtained via the INCell Analyzer, to illustrate a decrease in cell and nuclear area (µm^2^) following 0.1 pM rapamycin treatment (TIFF 252 kb)
