# Supplementary figures and images for: A novel, integrated in vitro carcinogenicity test to identify genotoxic and non-genotoxic carcinogens using human lymphoblastoid cells
Source: Arch Toxicol. 2017 Nov 6;92(2):935–51. doi: 10.1007/s00204-017-2102-y (PMC5818597; doi:10.1007/s00204-017-2102-y)

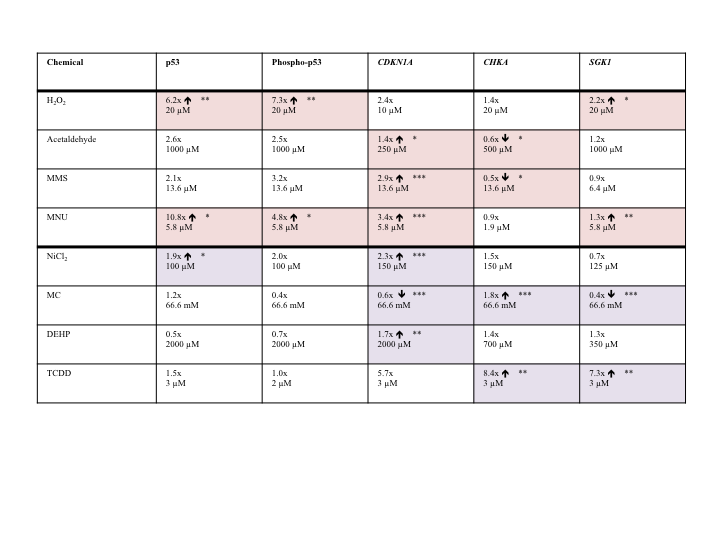

Supplement: Supplementary file 3 — Table summarising the fold-change results of cell signaling studies in response to chemical carcinogens. The concentration inducing the maximum significant effect is displayed. Arrows indicate the direction of change relative to the control and are only included for statistically significant results. If no significant change was observed, the lowest concentration inducing a quantitative change is displayed (TIFF 1521 kb) [file 204_2017_2102_MOESM3_ESM.tiff]

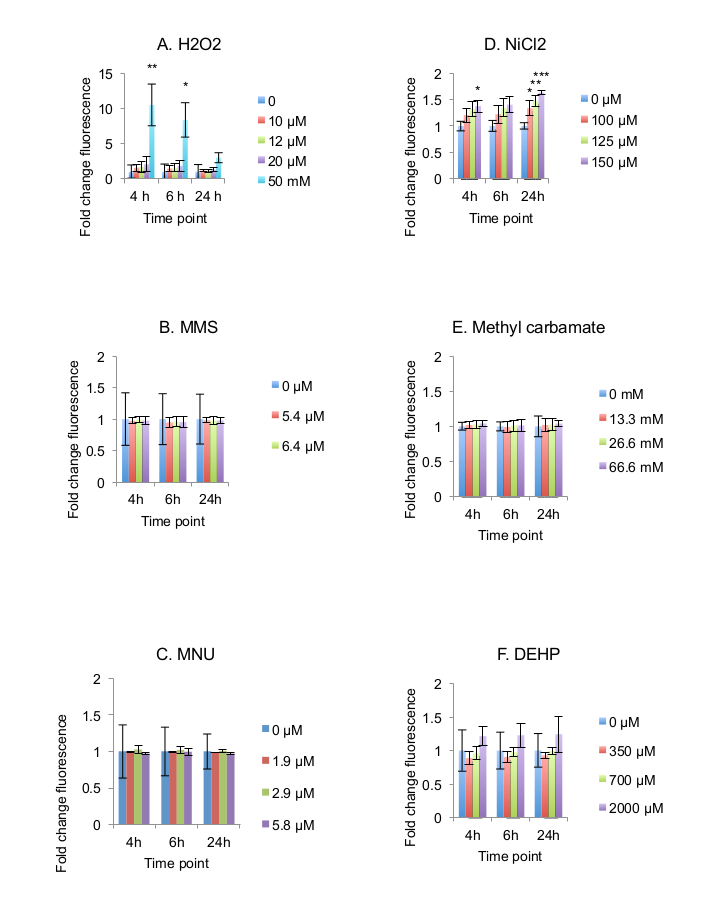

Supplement: Supplementary file 4 — Reactive oxygen species levels were studied using a standard DCFDA methodology. Readings of treated cells were taken at 4 h, 6 h and 24 h. H2O2 and NiCl2 produced significant increases in ROS evolution (TIFF 138 kb) [file 204_2017_2102_MOESM4_ESM.tiff]

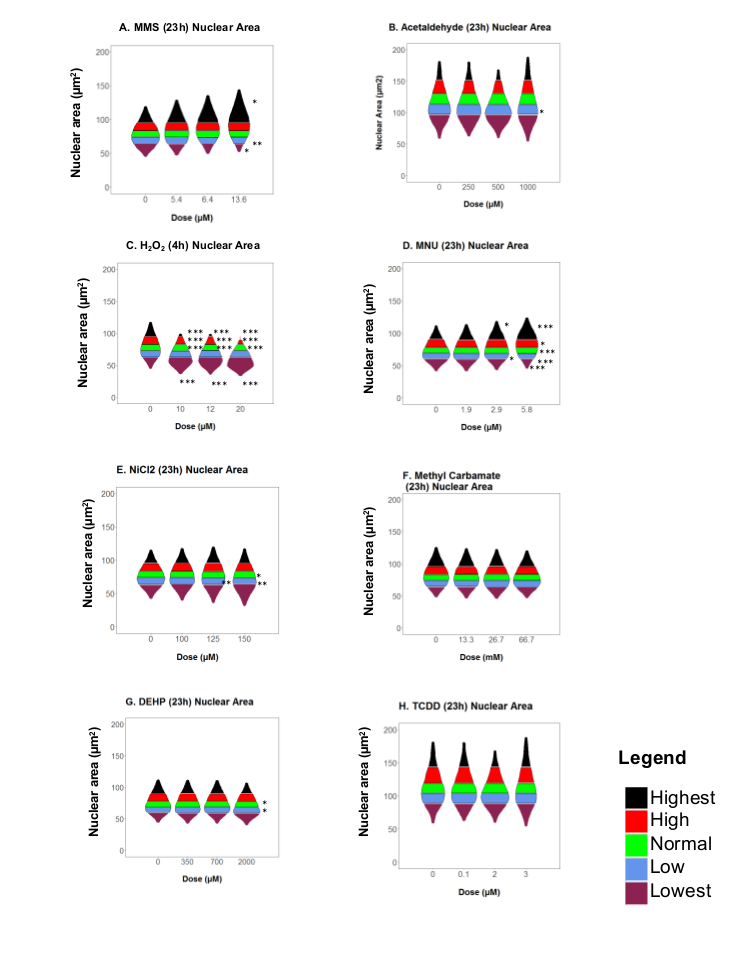

Supplement: Supplementary file 5 — Violin plots displaying nuclear area changes from data obtained via the INCell Analyzer, followed by Matlab-based image analysis. The frequency of cells (%) in each quintile category is plotted. Statistically significant changes in percentage cells relative to the vehicle control are denoted by *, where * = p ≤ 0.05, ** = p ≤ 0.01 and *** is p ≤ 0.001 (TIFF 178 kb) [file 204_2017_2102_MOESM5_ESM.tiff]

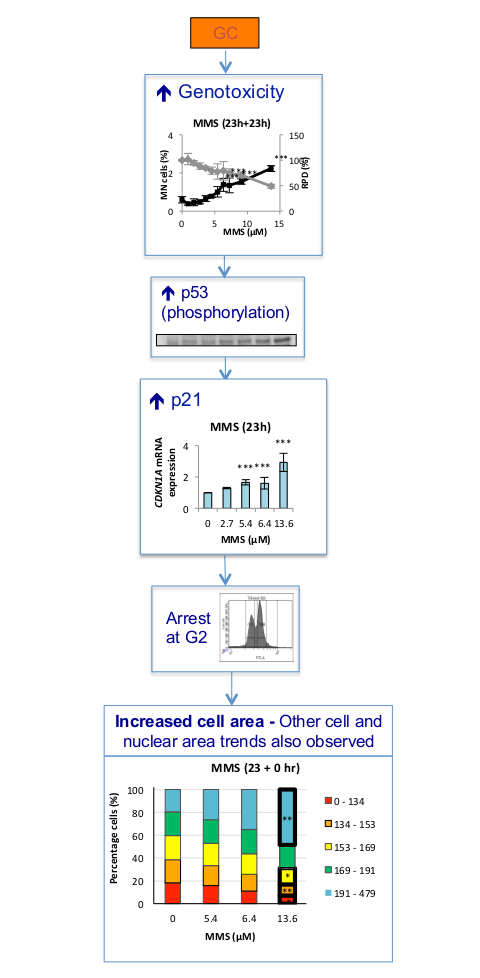

Supplement: Supplementary file 6 — Flow diagram illustrating the holistic nature of the “adverse outcomes” studied, based on the general results. Blue outlines indicate a series of events primarily associated with GCs, while green outlines indicate NGC-associated events. Orange indicates events that may be involved in either carcinogenic mechanism. Extracts from figures are included for illustrative purposes. An alternative, bar chart-based method of presenting the cell morphology data is indicated(TIFF 100 kb) [file 204_2017_2102_MOESM6_ESM.tiff]

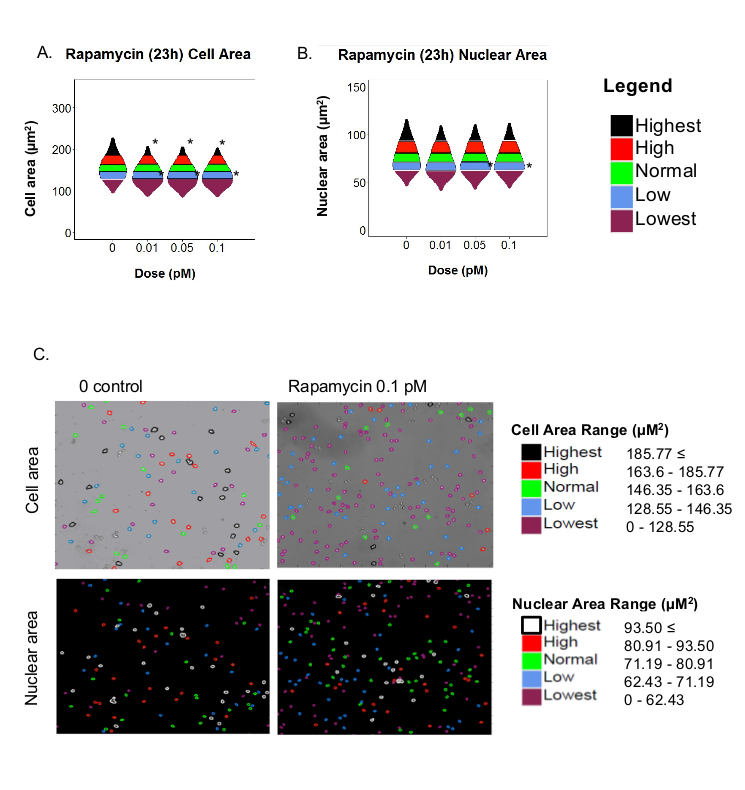

Supplement: Supplementary file 7 — Cell and nuclear data obtained using the INCell Analyzer 2000 for mTORC1 inhibitor, rapamycin, in TK6 cells (n = 2). A. Rapamycin (23 h + 0 h treatment) induced a reduction in cell area (n = 2), in agreement with previous observations (Fingar and Blenis, 2004). B. A similar reduction in nuclear area was observed in response to 23 h treatment with non-genotoxic carcinogen methyl carbamate. Asterisks represent p < 0.05. The concentrations included those inducing up to 50% cytotoxicity to limit non-chemical specific secondary toxicity effects. C. Colour-coded cell and nuclear perimeters overlaid on randomly selected raw images obtained via the INCell Analyzer, to illustrate a decrease in cell and nuclear area (µm2) following 0.1 pM rapamycin treatment (TIFF 252 kb) [file 204_2017_2102_MOESM7_ESM.tiff]
